# Hypertension modifies the association between serum Klotho and chronic kidney disease in US adults with diabetes: a cross-sectional study of the NHANES 2007–2016

**DOI:** 10.1080/0886022X.2025.2498089

**Published:** 2025-05-05

**Authors:** Tao Hong, Zelong Lian, Chaojun Zhang, Weihuang Zhang, Zhennan Ye

**Affiliations:** a Department of Nephrology, Institute of Nephrology, and Guangdong Provincial Key Laboratory of Autophagy and Major Chronic Non-Communicable Diseases, and Key Laboratory of Prevention and Management of Chronic Kidney Disease of Zhanjiang City, Affiliated Hospital of Guangdong Medical University, Zhanjiang, China; b Department of Information Center, Affiliated Hospital of Guangdong Medical University, Zhanjiang, China

**Keywords:** Chronic kidney disease, Klotho, hypertension, diabetes mellitus, interaction

## Abstract

**Context:**

The association between serum soluble Klotho (sKlotho) and chronic kidney disease (CKD) in individuals with diabetes mellitus (DM) remains controversial, and the influence of hypertension on this association is inconclusive.

**Objective:**

This study aims to investigate the joint association of sKlotho and hypertension with CKD prevalence in adults with DM.

**Methods:**

This cross-sectional study included 3,302 adults with DM from the National Health and Nutrition Examination Survey (2007–2016). Multivariate logistic regression analysis stratified by hypertension was used to assess the association between sKlotho and CKD prevalence. Moreover, the interaction between hypertension and sKlotho on CKD was evaluated.

**Results:**

Among individuals with DM, a significant association between sKlotho levels and CKD prevalence was observed only in those with hypertension. CKD prevalence was significantly lower in individuals with high sKlotho (≥ 806 pg/mL) than in those with low sKlotho (< 806 pg/mL) [adjusted OR = 0.54 (95% CI: 0.41–0.72); *p* < 0.001]. Moreover, a significant interaction between hypertension and sKlotho on CKD prevalence was observed among adults with DM [Multiplicative scale: OR = 0.65 (95% CI: 0.42–0.99); RERI = −0.80 (95% CI: −1.49 to −0.10); AP = −0.51 (95% CI: −0.90 to −0.12); SI = 0.44 (95% CI: 0.30–0.66)].

**Conclusions:**

Among DM adults, hypertension modified the association between sKlotho levels and CKD prevalence. Both additive and multiplicative interactions were observed between hypertension and sKlotho levels on CKD. The causalities between hypertension, Klotho, and CKD in diabetic patients need further exploration, and underlying mechanisms warrants elucidation.

## Introduction

Chronic kidney disease (CKD) has substantially increased the burden on global healthcare systems. The prevalence of CKD among United State adults reached 14.0% in 2020 [1] and is present in at least 10% of the population worldwide [2]. Meanwhile, the prevalence of diabetes mellitus (DM) has risen dramatically during past decades. As approximately 25%–40% of individuals with DM develop CKD [3], DM has emerged as the leading cause of CKD [4–7]. Despite optimal glycemic control with guideline-directed medical therapy, the progression of CKD cannot be halted in some diabetic patients [8]. Therefore, exploring the risk factors of CKD in diabetic patients and revealing their interactions may have great clinical significance for preventing the disease progression.

The Klotho gene, first identified in 1997 as a novel longevity gene [9], encodes α-Klotho, a transmembrane protein consisting of 1,012 amino acids with two extracellular domains and one intracellular domain. The extracellular portion can be cleaved by membrane proteases to form a soluble form of Klotho [10]. Klotho is primarily expressed in the distal convoluted tubules of the kidney, the choroid plexus, and the parathyroid glands, where it acts as a co-receptor of fibroblast growth factor-23 (FGF23) [11,12]. It exerts diverse renal protective effects, including anti-fibrosis, anti-oxidative stress, anti-inflammation, anti-apoptosis actions [13,14], while also modulating autophagy [15]. Klotho deficiency is a common feature of kidney diseases and plays a pivotal role in the pathogenesis and development of CKD [16,17].

Clinical studies investigating the relationship between circulating soluble Klotho (sKlotho) levels and CKD in diabetic patients have yielded inconsistent findings. A recent systematic review reported significantly lower sKlotho levels in patients with diabetic kidney disease (DKD) compared to diabetic patients without CKD [18]. Conversely, another study found that sKlotho levels were higher in DKD patients with an estimated glomerular filtration rate (eGFR) < 60 mL/min [19]. These discrepancies may be attributed to confounding factors, such as variations in the characteristics of study population.

Hypertension, a well-recognized risk factor of CKD [20,21], is one of the most common comorbidities of diabetes [22]. A recent meta-analysis confirmed hypertension as a factor significantly associated with CKD risk among type 2 diabetic patients [23]. Meanwhile, in clinical practice, those diabetic patients without hypertension still have chance to develop CKD. Besides, current evidence suggests that sKlotho deficiency predispose to hypertension by affecting key pathological pathways, including salt sensitivity, vascular calcification, arterial stiffness, endothelial dysfunction, and hyperaldosteronism [24–30]. As yet, although the association between sKlotho and CKD has been focused on and studied, the effects of downstream or regulatory factors of sKlotho on this association are rarely considered. Therefore, we ask if hypertension modifies the relationship between sKlotho and CKD and if there is an interaction between hypertension and sKlotho on CKD in DM patients.

This study aimed to evaluate the potential effect of hypertension on the association between sKlotho levels and the CKD prevalence in diabetic individuals using a nationally representative sample of the U.S. population.

## Methods

### Data sources

The National Health and Nutrition Examination Survey (NHANES) is a series of complex, stratified, multistage, continuous, and nationally representative surveys on the health and nutritional status of the non-institutionalized civilian populations in the United States (US). Detailed information is available in the NHANES survey methods and analytic guidelines [31]. Through home interviews, NHANES was provided obtained rich information on a range of health topics, including demographics, socioeconomic status, diet, health-related issues, and prescription medications, followed by blood sampling at a mobile examination center (MEC) [31]. NHANES was reviewed and approved by the National Center for Health Statistics (NCHS) Research Ethics Review Board, and obtained consent from all participants. NHANES datasets are publicly available at https://www.cdc.gov/nchs/nhanes/index.htm.

### Study design and participants

**Figure 1.**
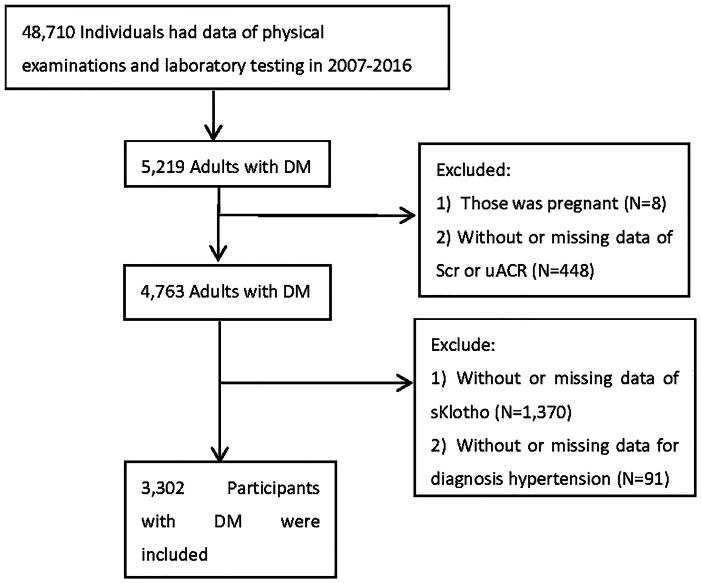
Flow chart of the screening and enrollment of study participants. Abbreviations: DM, diabetes mellitus; Scr, serum creatinine; uACR,urinary albumin- creatinine ratio.

For this analysis, data from 5 continuous NHANES cycles (2007–2008, 2009–2010, 2011–2012, 2013–2014, and 2015–2016) were combined. During this period, 48,710 individuals from various regions across the US underwent physical examinations and laboratory testing. Among them, 5,219 adults with DM were identified. SKlotho levels were measured exclusively in individuals aged 40–79 years who consented to the use of surplus serum in future research. Individuals lacking data on important variables (serum creatinine, urinary albumin-creatinine ratio [uACR], sKlotho, or data for diagnosing hypertension) were excluded. Finally, a total of 3,302 participants with DM were included in the analysis (Figure 1).

### Definition of DM

NHANES officials asked participants if they had been told they had DM by a medical professional, and those who answered affirmatively were considered to have DM. In addition, individuals who met any one of the following criteria were also considered to have DM: 1) Glycated hemoglobin, type A1C (HbA1c) ≥ 6.5%; 2) fasting glucose ≥ 7.0 mmol/L; 3) random blood glucose ≥ 11.1 mmol/L; 4) 2-h oral glucose tolerance test (OGTT) blood glucose ≥ 11.1 mmol/L; or 5) use of hypoglycemic drugs [32]. The hypoglycemic drugs included insulin and oral hypoglycemic drugs (acarbose, glimepiride, glipizide, linagliptin, metformin, sitagliptin, nateglinide, pioglitazone, repaglinide, and rosiglitazone).

### Study variables

The main exposure variable was the sKlotho level. The effect modifier variable was hypertension.

### Measurement of sKlotho levels

SKlotho levels in frozen serum samples collected between 2007 and 2016 were evaluated between 2019 and 2020. Pristine serum samples obtained from participants aged 40–79 years who provided consent for use in future research, were stored at a temperature of −80 °C. The samples were then analyzed using the sandwich ELISA test (IBL-International, Japan) at the Northwest Lipid Metabolism and Diabetes Research Laboratories, University of Washington. All sample analyses were performed in duplicate, and the average of the two values was used to calculate the final value, according to the manufacturer’s protocol. The results were checked to meet the laboratory’s standardized criteria for acceptability. This assay is reported to has a sensitivity of 6 pg/mL [33]. Several dilutions of the two samples with high or extremely high Klotho concentrations were used to evaluate the linearity of the assay. The linearity of the plots of the expected vs the observed values was exceptional. No cutoff values have been specified for sKlotho. Additional information is available on the NHANES website.

### Definition of hypertension

Three times of blood pressure measurements, including systolic and diastolic blood pressures, using a mercury sphygmomanometer, were recorded at the MEC or at home. The measurements were performed by NHANES examiners. The average of the three blood pressure measurement values were adopted as the participant’s blood pressure number. The diagnostic criteria for hypertension are as follows: systolic blood pressure ≥ 140 and/or diastolic blood pressure ≥ 90 mmHg, or self-reported physician diagnosis and/or self-reported currently using of anti-hypertensive medications (Considering that RAS-inhibitors are often used to lower urinary protein and protect kidney rather than lower blood pressure in clinical practice, using of RAS-inhibitors alone was not regarded as the diagnostic criteria.).

### Assessment of outcomes

The primary outcome was CKD. According to the Kidney Disease: Improving Global Outcomes (KDIGO) 2021 Clinical Practice Guideline for the Management of Glomerular Diseases, CKD is classified based on the cause, GFR category (G1–G5), and albuminuria category (A1–A3) [34]. Because most people had only one measurement in the survey and the timing of the second urine sample collection differed when repeat proteinuria was measured in selected NHANES cycles or subsamples, a one-time uACR was used instead of 24-h persistent proteinuria to reduce bias, as previously described [35–37]. In addition, according to the previous literature, the Chronic Kidney Disease Epidemiology Collaboration 2021 (CKD-EPI 2021) equation was used to estimate GFR [38], named eGFR.

The eGFR was calculated using the Chronic Kidney Disease Epidemiology Collaboration equation (CKD-EPI 2021) as follows:

$$\begin{array}{c} \text{eGFR}=142\times \text{min }\left(\text{Scr}/\kappa ,\text{ 1}\right)\text{α}\times \text{max }\left(\text{Scr}/\kappa ,\text{ 1}\right)\\ -1.200\times 0.\text{9938Age}\times 1.012\;\left[\text{if female}\right], \end{array}$$

in which Scr is standardized serum creatinine in mg/dL, κ is 0.7 for females and 0.9 for males, α is −0.241 for females and −0.302 for males, min means the minimum of Scr/κ or 1, and max means maximum of Scr/κ or 1. The eGFR units are mL/min/1.73 m^2^.

The uACR was calculated using the following equation: uACR (mg/g) = urinary albumin (mg/dL)/urinary creatinine (g/dL).

Patients who met any one of the following conditions were classified as having CKD: (1) eGFR < 60 mL/min/1.73 m^2^; and/or (2) uACR ≥ 30 mg/g [39].

### Covariates

By reviewing previous literatures, the clinical meaningful variables or important risk factors reported before were considered as candidates for confounders. Then, the clinical meaningful variables or ones that affected the regression coefficients by more than 10%, were selected as confounders to adjust in the final model. Demographics included age, sex, and race (non-Hispanic White, non-Hispanic Black, Mexican American, and other Race). Socioeconomic variables included education level (less than high school, high school or general educational development, above high school), the ratio of income-poverty (categorical, ≤ 1.3 as low, 1.3–3.5 as middle, > 3.5 as high) [40–43]. Lifestyle variables included marital status (married or living with partner, living alone), body mass index (BMI)(< 28 kg/m^2^, ≥ 28 kg/m^2^), smoking status (never smoker, former smoker, and current smoker), drinking habits (never drinker, former drinker, and current drinker), physical activity (inactive group with MET-min/week < 600, insufficiently active group with MET-min/week between 600 to 1800, and active group with MET-min/week ≥ 1800), and CVD history (yes or no) [40–43]. Laboratory examinations included evaluation of total cholesterol, triglycerides, serum calcium, serum phosphorus, aspartate transaminase (AST), alanine transaminase (ALT), pulse pressure (<60 mmHg, ≥60 mmHg), and glycated hemoglobin [40–43]. Prescription medications included statins, insulin, and renin-angiotensin-aldosterone system inhibitors (RAS-inhibitors) (enalapril, lisinopril, benazepril, fosinopril, quinapril, ramipril, captopril, perindopril, moexipril, trandolapril, losartan, olmesartan, valsartan, telmisartan, irbesartan, candesartan, spironolactone, aliskiren, and eplerenone).

In our study, patients who self-reported being diagnosed with cardio-vascular disease (CVD) by their physician were defined as having CVD history.

### Statistics analysis

All statistical analyses were performed in accordance with the CDC guidelines [31]. Considering that NHANES employs a complex probability sample design and oversamples some population groups to ensure adequate representation, sample weights were utilized to combine the survey cycles and estimate the mean values and standard deviation (SD).

Participants were grouped by their sKlotho levels and diagnosis of hypertension, and the baseline characteristics of the participants were described across groups. Continuous variables are represented by the mean ± SD or median (interquartile range, IQR), and categorical parameters are expressed as numbers and percentage frequencies (%).

Restricted cubic spline analysis was used to examine the presence of a nonlinear relationship between sKlotho levels and CKD prevalence in hypertension and non-hypertension groups. Given that the significant inflection point of CKD prevalence was near the median level of sKlotho (806 pg/mL) in the fitting curve (Figure 2), the median sKlotho level was used as the threshold, based on which participants were grouped to low and high sKlotho groups.

Figure 2.Restricted cubic spline analysis evaluating the adjusted or of CKD prevalence across the distribution of sKlotho in group of hypertension (A) and group of non-hypertension (B). *Note:* The adjusted model included the following covariates: age, sex, race, education levels (less than high school, high school or equivalent, and college or above), marital status (have partner or no), income level (PIR < 1.3, 1.3 ≤ PIR < 3.5, or PIR≥ 3.5), drinking status (never drinker, former drinker, or current drinker), smoking status (never smoker, former smoker, or current smoker), BMI (< 28 kg/m^2^ or ≥ 28 kg/m^2^), physical activity (inactive group, insufficiently active group, and active group), CVD history (yes or no), total cholesterol (continuous), triglyceride (continuous), serum calcium (continuous), serum phosphorus (continuous), alanine transaminase (continuous), aspartate transaminase (continuous), pulse pressure (< 60mmHg, or ≥ 60mmHg), glycated hemoglobin (continues), using RAS-inhibitors (yes or no), using statins (yes or no), and using insulin (yes or no).

**Figure:**
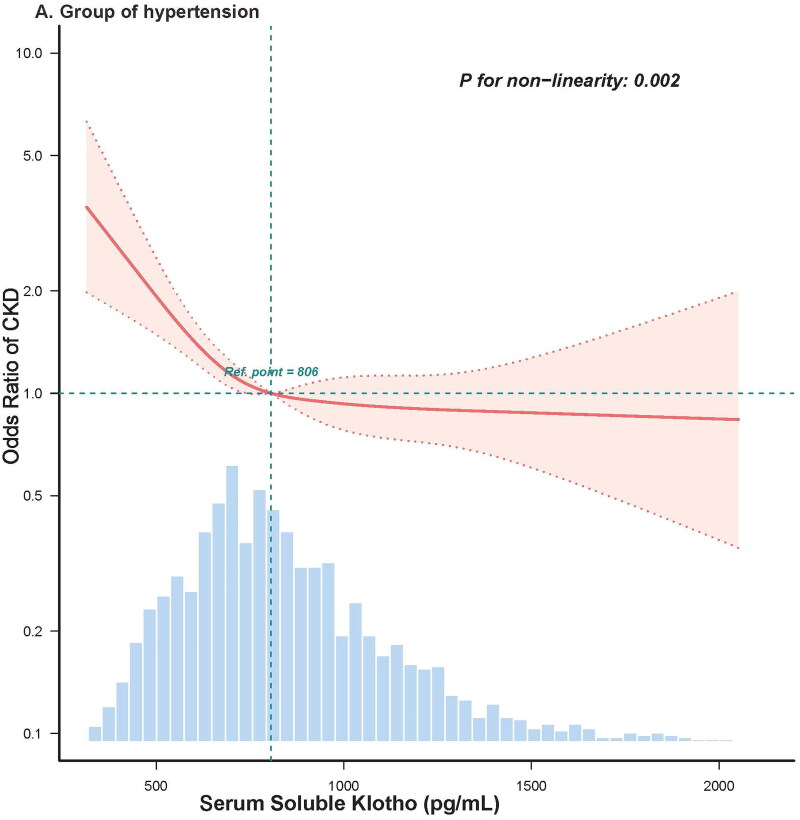

**Figure:**
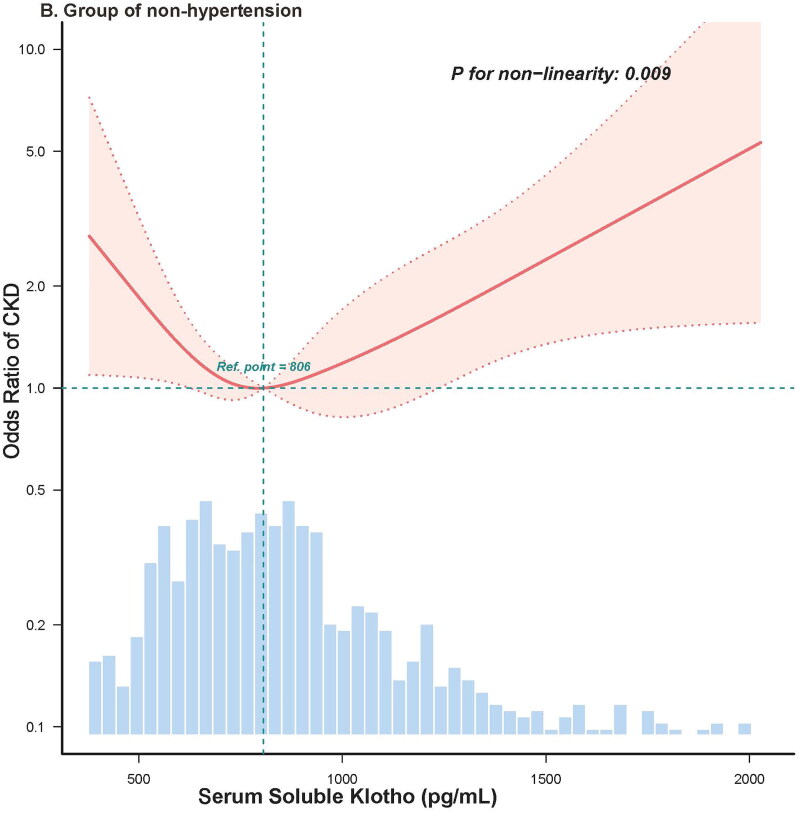

Sampling-weighted multivariable logistic regression analyses were performed to examine the association between sKlotho levels and CKD prevalence in different models. The sKlotho level was analyzed as a categorical (low and high) variable. Model 1 was adjusted for age, biologic sex, race, education level, marital status, and income. Model 2 included model 1 and CVD history, drinking habits, smoking habits, physical activity, and BMI. Model 3 included Model 2 plus total cholesterol, triglycerides, serum calcium, serum phosphorus, ALT (alanine transaminase), AST (aspartate transaminase), pulse pressure, and glaciated-hemoglobin. Model 4 was established from Model 3 plus drug prescription with RAS-inhibitors, statins, and insulin.

The interaction between hypertension and sKlotho on CKD prevalence and its components was evaluated on both additive and multiplicative scales [44–46]. The interaction on the multiplicative scale indicates whether the risk of the outcome for individuals with both risk factors differed significantly from the product of the outcome risks for individuals with only 1 risk factor. Multiplicative interaction was evaluated by adding an ‘sKlotho * hypertension’ term into the regression models. Additive interaction represents that the coexistence of both risk factors has a significantly greater or smaller risk than the sum of the independent risk of each risk factor. Interaction on the additive scale was investigated by calculating the relative excess risk due to interaction (RERI), the attributable proportion due to interaction (AP) and synergy index (S index) according to the algorithm released by Andersson et al. [44]. RERI was calculated as (OR11 - OR10 - OR01) + 1, where OR11 is the outcome risk for the coexistence of high sKlotho levels and hypertension, and OR10 indicates the risk for high sKlotho levels without hypertension, and OR01 represents the risk for low sKlotho levels and hypertension. AP was determined as RERI/OR11 and represented the proportion of the total risk attributed to the interaction. Finally, the S index was calculated as [OR11 − 1]/[(OR10 − 1) + (OR01 − 1)]. For the RERI and AP, a 95% CI not including 0 indicated significance, and for the S-index, a 95% CI not including 1 indicated significance.

Distributions of missing data for all covariates are shown in Table S1. Because the proportion of missing data was less than 10%, participants with missing covariate data were excluded from the multivariate adjusted model analysis.

Sensitivity analysis was performed to test the robustness of our findings. First, stratified analyses were conducted by age, sex, smoking habit, BMI, and triglycerides. Second, a sensitivity analysis using multiple imputation was performed to maximize statistical power and minimize bias that might occur account for missing data.

All statistical analyses were performed using the statistical software packages R 4.3.2 (http://www.R-project.org, The R Foundation) and Free Statistics software version 1.9.2. A two-tailed test was performed, and statistical significance was set at *p* < 0.05.

## Results

### Characteristics of the participants

Five cycles of NHANES data (2007–2016) were used in this study. After excluding individuals with missing data on important variables, a total of 3,302 adult participants with DM were included in the final analyses. The flowchart of the exclusion criteria is shown in Figure 1. The baseline characteristics of 3,302 participants stratified by diagnosis of hypertension (yes *vs* no) and sKlotho levels (< 806 pg/mL *vs* ≥ 806 pg/mL) are shown in Table 1, representing approximately 20.318 million US adults aged ≥ 40 years. Participants with high sKlotho levels and without hypertension were more likely to be younger and with a relatively lower prevalence of CVD history, hyperuricemia, high pulse pressure, and CKD. Compared with participants without hypertension, those with hypertension had less physical activity, higher levels of BMI, triglycerides, uACR, serum uric acid, pulse pressure, and more proportions of using RAS-inhibitors and statins. No obvious difference in levels of education, income, serum calcium, serum phosphorus, or serum albumin was observed among these groups.

**Table 1.** Baseline characteristics of 3,302 patients with DM grouped by sKlotho level and hypertension.

Variables	All (*N* = 3,302)	Non-hypertension (*n* = 836)		Hypertension (*n* = 2,466)		*P-*value
		sKlotho < 806 (pg/mL) (*n* = 385)	sKlotho ≥806 (pg/mL) (*n* = 451)	sKlotho < 806 (pg/mL) (*n* = 1,265)	sKlotho ≥ 806 (pg/mL) (*n* = 1,201)	
Sex, *n* (%)						0.233
Male	1,699 (52.71)	211 (54.12)	249 (55.87)	666(54.24)	573(49.00)	
Female	1,603 (47.29)	174 (45.88)	202 (44.13)	599(45.76)	628(51.00)	
Age (years)	60.13 ± 10.15	57.40 ± 10.63	55.80 ± 9.93	62.44 ± 9.77	60.49 ± 9.61	< 0.001
Race, *n* (%)						< 0.001
Non-Hispanic White	1,130 (63.47)	143 (61.86)	156 (64.46)	467(66.53)	364(60.15)	
Non-Hispanic Black	767 (12.61)	49 (7.30)	60 (6.94)	318 (13.48)	340 (16.17)	
Mexican American	668 (9.94)	107 (13.90)	113 (12.89)	223(7.63)	225(9.74)	
Other Race	737 (13.98)	86 (16.94)	122 (15.71)	257(12.36)	272(13.94)	
Education, *n* (%)						0.6222
< 9th grade	640 (10.62)	86 (11.38)	87 (9.05)	239 (9.94)	228 (11.83)	
9–12 grades	1,311 (36.71)	147 (34.33)	168 (36.48)	519 (38.64)	477 (35.51)	
≥ 12 years	1,349 (52.67)	152 (54.29)	196 (54.47)	505 (51.43)	496 (52.66)	
marital status, *n* (%)						0.1214
Have partner	2,057 (67.02)	262 (72.74)	295 (69.03)	770 (66.68)	730 (64.33)	
living alone	1,243 (32.98)	123 (27.26)	156 (30.97)	493(33.32)	471(35.67)	
Income, *n* (%)						0.2608
PIR < 1.3	1,131 (25.20)	131 (22.23)	143 (20.21)	439 (25.90)	418 (27.76)	
1.3 ≤ PIR < 3.5	1,168 (37.80)	133 (39.60)	162 (37.28)	444 (38.36)	429 (36.75)	
PIR ≥ 3.5	716 (37.00)	84 (38.16)	110 (42.51)	267(35.74)	255 (35.49)	
CVD history, *n* (%)						< 0.001
No	2,654 (80.60)	332 (86.60)	407(89.73)	955 (77.68)	960 (77.55)	
Yes	648 (19.40)	53 (13.40)	44 (10.27)	310(22.32)	241(22.45)	
Drinking habit, *n* (%)						0.5047
Never	565 (15.15)	60 (13.64)	72 (15.39)	216 (15.29)	217 (15.44)	
Former	922 (26.62)	101 (21.86)	127 (24.50)	368 (27.13)	326 (28.77)	
Current	1,625 (58.23)	204 (64.50)	218 (60.11)	615 (57.58)	588 (55.79)	
Smoking habit, *n* (%)						0.2340
Never	1,598 (47.28)	180 (44.71)	225(52.47)	557 (45.20)	636(48.28)	
Former	1,133 (36.32)	127 (36.85)	137 (29.21)	483 (39.23)	386 (36.00)	
Current	568 (16.41)	78 (18.44)	89 (18.32)	223(15.57)	178 (15.72)	
Physical activity, *n* (%)						0.009
MET-min/wk ≤ 600	1,835 (53.14)	194 (45.47)	216 (45.70)	749 (57.53)	676 (54.37)	
600 < MET-min/wk ≤ 1800	616 (20.12)	86 (25.14)	103 (25.28)	215 (17.84)	212 (18.50)	
MET-min/wk > 1800	851 (26.74)	105 (29.38)	132 (29.02)	301(24.62)	313 (27.13)	
BMI (Kg/m^2^)	32.95 ± 7.06	31.19 ± 6.26	31.53 ± 6.79	33.38 ± 6.85	33.78 ± 7.50	< 0.001
BMI < 28, *n* (%)	933 (25.58)	141 (35.85)	171 (34.51)	324 (23.15)	297 (20.39)	
BMI ≥ 28, *n* (%)	2,317 (74.42)	237 (64.15)	278 (65.49)	914 (76.85)	888 (79.61)	
Pulse pressure, *n* (%)						< 0.001
≥ 60 mmHg, *n* (%)	1,447 (40.67)	96 (20.38)	102 (19.78)	668 (52.95)	581 (45.84)	
Using Medications, *n* (%)						
RAS-inhibitors	1,851 (57.34)	105 (27.67)	103 (22.32)	886 (72.98)	757 (66.67)	< 0.001
Statins	1,611 (51.17)	158 (46.61)	149 (36.59)	719 (58.57)	585 (51.04)	< 0.001
Insulin	559 (17.70)	50 (12.25)	60 (13.48)	253 (21.42)	196 (17.44)	< 0.001
Triglycerides (mmol/L)	1.85 [1.23, 2.74]	1.78[1.20, 2.48]	1.75 [1.19, 2.81]	1.89[1.25, 2.65]	1.90[1.23, 2.82]	0.4504
Cholesterol (mmol/L)	4.795 ± 1.193	4.89 ± 1.32	4.97 ± 1.22	4.68 ± 1.17	4.81 ± 1.14	0.0067
Serum calcium (mmol/L)	2.359 ± 0.095	2.36 ± 0.09	2.35 ± 0.08	2.36 ± 0.10	2.36 ± 0.10	0.1555
Serum phosphorus (mmol/L)	1.203 ± 0.182	1.19 ± 0.17	1.20 ± 0.19	1.21 ± 0.19	1.20 ± 0.18	0.5040
Serum folate (nmol/L)	42.2 [28.1, 63.6]	42.47 [29.0, 59.64]	41.93[27.9, 60.55]	43.94 [27.90, 68.95]	40.80[27.65, 61.50]	0.2212
Serum uric acid (μmol/L)	333.1 [279.6, 398.5]	315.20 [267.70, 380.70]	309.3 [255.80, 356.90]	356.90[297.40, 416.40]	333.1[285.50, 392.60]	< 0.001
Hyperuric, *n* (%)						< 0.001
No	2,399 (73.26)	299 (78.73)	393 (88.29)	825 (65.97)	882(72.73)	
Yes	901 (26.74)	86 (21.27)	58 (11.71)	440 (34.03)	317 (27.27)	
Serum albumin (g/L)	42.0 [40.0, 44.0]	42.0 [40.0, 44.0]	42.0 [40.0, 44.0]	42.0 [40.0, 44.0]	42.0 [40.0, 44.0]	0.1050
AST (U/L)	27.59 ± 19.38	25.46 ± 12.96	29.78 ± 37.16	26.00 ± 11.62	29.22 ± 16.33	< 0.001
ALT (U/L)	28.29 ± 22.76	26.31 ± 12.50	32.46 ± 40.95	25.84 ± 13.92	29.96 ± 22.0	< 0.001
uACR (mg/g)	10.65 [5.97, 28.82]	7.41[5.12, 14.61]	8.42 [5.08, 19.03]	12.88 [7.07,41.41]	10.91[6.54, 30.93]	< 0.001
Glycated hemoglobin (%)	6.70 [6.00, 7.70]	6.50 [5.90,7.60]	6.70 [6.00, 8.20]	6.60 [6.00, 7.40]	6.80 [6.20, 8.00]	< 0.001
Hemoglobin (g/dL)	14.08 ± 1.51	14.21 ± 1.45	14.59 ± 1.42	13.84 ± 1.57	14.08 ± 1.46	< 0.001
eGFR (mL/min/1.73 m^2^)	85.01 ± 21.63	90.59 ± 19.311	93.36 ± 16.95	78.70 ± 23.38	86.33 ± 20.14	< 0.001
CKD, *n* (%)						< 0.001
No	2,108 (68.51)	301 (80.69)	349 (80.98)	681 (58.75)	777 (69.41)	
Normoal-albuminuric CKD	255 (7.31)	22 (4.96)	12 (2.89)	148 (11.61)	73 (5.29)	
Albuminuric CKD	939 (24.18)	62 (14.35)	90 (16.13)	436 (29.64)	351 (25.30)	

***Abbreviations:*** ALT, alanine transaminase; AST, aspartate transaminase; BMI, body mass index; CKD, chronic kidney disease; CVD, cardiovascular disease; eGFR, estimated glomerular filtration rate; MET-min/wk, metabolic equivalent minutes each week; PIR, ratio of family income-poverty; uACR, urinary albumin-creatinine ratio; RAS, renin-angiotensin-aldosterone system.

### Hypertension modifies the association between sKlotho and CKD prevalence

To explore whether hypertension affected the association between sKlotho levels and CKD prevalence, diabetic participants were stratified according to the diagnosis of hypertension (hypertension and non-hypertension groups). In the hypertension group, the restricted cubic spline fitting curve showed a nonlinear relationship between sKlotho and CKD prevalence (*P*-nonlinear = 0.002), with inflection points for CKD prevalence near the median of sKlotho (806 pg/mL) (Figure 2A). The prevalence of CKD decreased with an increase in sKlotho level, although the decreasing trend slowed down when sKlotho exceeded 806 pg/mL, displaying an L-shaped pattern (Figure 2A). In the group of non-hypertension, a significant nonlinear relationship was also observed between sKlotho levels and CKD prevalence (*P*-nonlinear = 0.009). However, the fitting curve revealed a V-shaped pattern showing that, as sKlotho levels increased, the CKD prevalence initially decreased until sKlotho levels exceeded 806 pg/mL and increased above this threshold (Figure 2B).

In weighted multivariate logistic regression analyses stratified by hypertension, the ORs and corresponding 95% CIs for CKD prevalence according to sKlotho levels as a categorical variable (sKlotho < 806 pg/mL, and klotho ≥ 806 pg/mL) are summarized in Table 2. No significant association between sKlotho levels and CKD prevalence was observed in participants without hypertension (Table 2). However, among participants with hypertension, higher sKlotho (≥ 806 pg/mL) was significantly associated with an decreased prevalence of CKD in the non-adjusted model (OR = 0.62; 95% CI: 0.51–0.76, *p* < 0.001). This association remained stable in the fully adjusted model (OR = 0.54; 95% CI: 0.41–0.72, *p* < 0.001), indicating a 46% decrease in CKD prevalence than lower sKlotho (<806 pg/mL) (Table 2).

**Table 2.** Weighted multivariable logistic regression models evaluating the association between sKlotho and CKD prevalence.

			Non-adjusted Model		Model 1		Model 2		Model 3		Model 4	
Variable	*n*. total	*n*. event (%)	OR (95%CI)	*P*-value	OR (95%CI)	*P*-value	OR (95%CI)	*P*-value	OR (95%CI)	*P*-value	OR (95%CI)	*P*-value
Group of hypertension												
sKlotho < 806 pg/mL	1,265	584 (46.5)	ref		ref		ref		ref		ref	
sKlotho ≥ 806 pg/mL	1,201	424 (35.3)	0.62 (0.51, 0.76)	**<0.001**	0.65 (0.52, 0.81)	**<0.001**	0.63 (0.49, 0.80)	**<0.001**	0.54 (0.41, 0.72)	**<0.001**	0.54 (0.41, 0.72)	**<0.001**
Group of non-hypertension												
sKlotho < 806 pg/mL	385	84 (21.8)	ref		ref		ref		ref		ref	
sKlotho ≥ 806 pg/mL	451	102 (22.6)	0.99 (0.69, 1.41)	0.947	1.05 (0.73, 1.49)	0.802	1.07 (0.74, 1.54)	0.724	0.85 (0.56, 1.28)	0.431	0.94 (0.62, 1.42)	0.752

*Notes:* Model 1 adjusts for age, sex, race, education level, marital status (have partner or no), and income level; Model 2 adjusts for adjusts I + CVD history + drinking habits + smoking habits + physical activity + BMI; Model 3 adjusts for adjusts 2 + triglyceride + total cholesterol + serum calcium + serum phosphorus + Glycated hemoglobin + ALT+ AST + pules pressure; Model 4 adjusts for adjusts 3 + using RAS-inhibitors + using statins + using insulin.

### Interaction of sKlotho and hypertension on CKD prevalence

**Figure 3.**
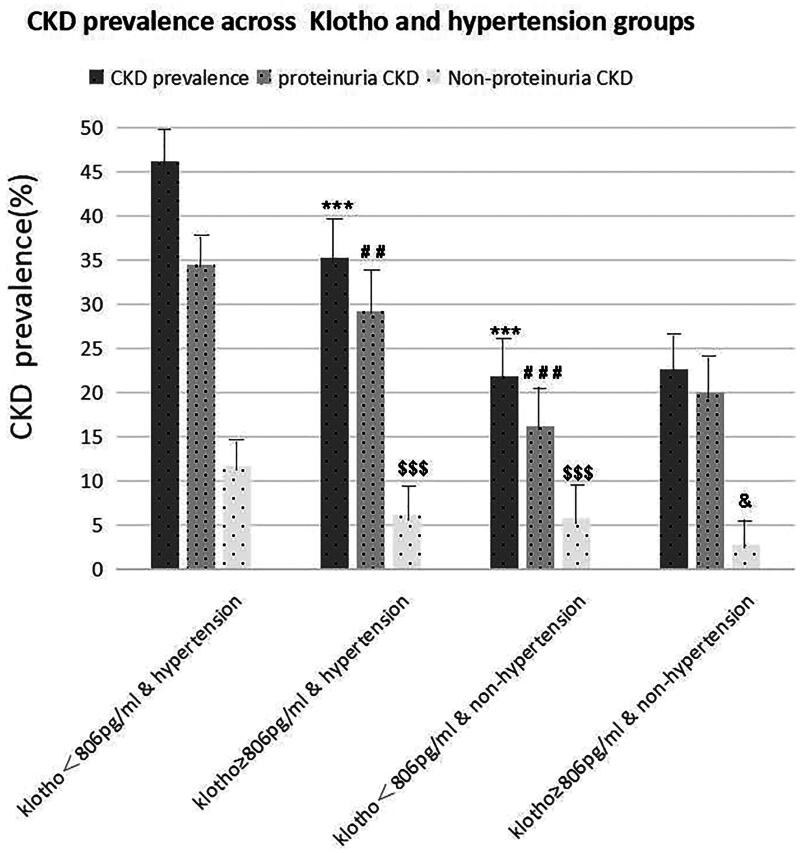
CKD prevalence in different sKlotho levels and hypertension groups. Asterisks represent differences compared to CKD prevalence with Klotho < 806 pg/mL & hypertension (***P < 0.001); compared to proteinuria CKD with Klotho < 806 pg/mL & hypertension (^##^*P* < 0.01, ^###^*P* < 0.001); compared to non-proteinuria CKD with Klotho < 806 pg/mL & hypertension (^$$$^*P* < 0.001); compared to non-proteinuria CKD with Klotho < 806 pg/mL & non-hypertension group (^&^*P* < 0.05).

The prevalence of CKD was compared by classifying the participants into 4 groups: hypertension or none-hypertension combined with low or high sKlotho levels. The prevalence of CKD in different groups is shown in Figure 3. The highest CKD prevalence was observed in the group of low sKlotho levels and hypertension. The proportion of non-protenuria CKD was lowest in the group of high sKlotho levels and non-hypertension. Among participants with hypertension, the CKD prevalence was significantly higher in the group of lower sKlotho (*p* < 0.001). Between groups with lower sKlotho, the CKD prevalence was significantly higher in the group of hypertension (*p* < 0.001). However, among participants without hypertension, there was no significantly difference in CKD prevalence between groups of different sKlotho levels (*p* > 0.05) (Figure 3).

The interaction effect analysis indicated a significant antagonistic effect of sKlotho and hypertension on CKD in Model 4 (Table 3). For the interaction in the additive scale, the adjusted RERI (−0.80; 95% CI: −1.49 to −0.10) and adjusted AP (-0.51; 95% CI: −0.902 to −0.12) were negative, and the adjusted S index (0.41; 95% CI: 0.24–0.70) was <1, demonstrating the significant and antagonistic interaction between hypertension and sKlotho. Moreover, for the interaction in the multiplicative scale, the value of the adjusted multiplicative scale (0.65, 95% CI: 0.42–0.99) was < 1, demonstrating the significant and antagonistic multiplicative interaction between hypertension and sKlotho levels (Table 3).

**Table 3.** Interaction effect analysis of hypertension and sKlotho on CKD prevalence.

Klotho (low/high)	Hypertension (no/yes)	CKD / Total (N)	Non-adjusted		Model 1		Model 2		Model 3		Model 4	
			OR (95%CI)	*P* value	OR (95%CI)	*P* value	OR (95%CI)	*P* value	OR (95%CI)	*P* value	OR (95%CI)	*P* value
0	0	385 (84)	1.0		1.0		1.0		1.0		1.0	
0	1	1,265 (584)	3.07 (2.36, 4.01)	0.0	2.58 (1.94, 3.44)	0.0	2.57 (1.9, 3.47)	0.0	2.24 (1.63, 3.07)	0.0	2.31 (1.67, 3.21)	0.0
1	0	451 (102)	1.05 (0.75, 1.45)	0.78	1.15 (0.81, 1.63)	0.43	1.16 (0.80, 1.67)	0.43	1.00 (0.69, 1.46)	1.00	1.03 (0.71, 1.51)	0.86
1	1	1,201 (424)	1.96 (1.49, 2.56)	0.0	1.75 (1.31, 2.34)	0.0	1.76 (1.30, 2.39)	0.0	1.47 (1.07, 2.02)	0.02	1.55 (1.12, 2.16)	0.01
Multiplicative scale (95% CI)			0.61 (0.42, 0.88)		0.59 (0.40, 0.87)		0.60 (0.41, 0.88)		0.66 (0.43, 1.00)		0.65 (0.42, 0.99)	
RERI (95% CI)			−1.16 (−1.87, −0.46)		−0.98 (−1.67, −0.29)		−0.96 (−1.68, −0.24)		−0.77 (−1.43, −0.10)		−0.8(−1.49, −0.1)	
AP (95% CI)			−0.60 (−0.90, −0.29)		−0.56 (−0.90, −0.22)		−0.55 (−0.90, −0.19)		−0.52 (−0.92, −0.13)		−0.51 (−0.9, −0.12)	
**SI**			0.45 (0.33, 0.62)		0.43 (0.30, 0.63)		0.44 (0.3, 0.66)		0.38 (0.21, 0.68)		0.41 (0.24, 0.7)	

*Notes:* Model 1 adjusts for age, sex, race, education level, marital status, and income level; Model 2 adjusts for adjusts I + CVD history + drinking habits + smoking habits + physical activity + BMI; Model 3 adjusts for adjusts 2 + triglyceride + total cholesterol + serum calcium + serum phosphorus + glycated hemoglobin + ALT+ AST+ pules pressure; Model 4 adjusts for adjusts 3 + using RAS-inhibitors + using statins + using insulin. Abbreviation: RERI: relative excess risk due to interaction; AP: attributable proportion due to interaction; SI: synergy index.

### Sensitivity analyses

Sensitivity analyses were performed using a multiple imputations to address missing data. The results of the multivariate logistic regression analyses using multiple imputation data were generally robust and are displayed in Supplementary Table S2.

**Figure 4.**
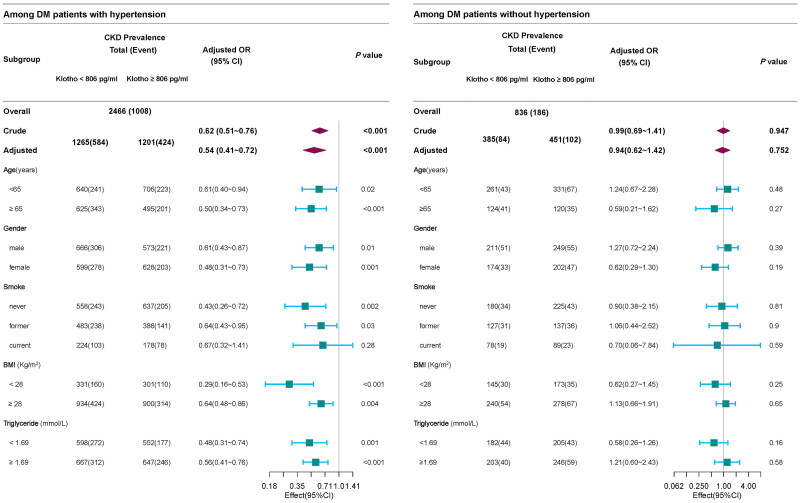
Subgroup analyses of the association between sKlotho and CKD in hypertension and non-hypertension groups, respectively. *Note:* The model adjusted following covariates: age, sex, race, education levels, marital status, income levels, drinking status, smoking status, BMI, physical activity, CVD history, total cholesterol, triglyceride, serum calcium, serum phosphorus, alanine transaminase, aspartate transaminase, pulse pressure, glycated hemoglobin, using RAS-inhibitors, using statins, and using insulin.

Furthermore, stratified analysis was performed to examine whether the interaction between hypertension and sKlotho on CKD were stable among the different subgroups. None of the variables, including age (<65 years), BMI (<28 kg/m^2^), or triglycerides (<1.69 mmol/L), significantly affected the association between sKlotho levels, and CKD in patients with and without hypertension, indicating the robustness and reliability of this association (Figure 4). However, among participants with hypertension, no significant association was observed between sKlotho and CKD in the subgroups of current smoker, indicating an interaction between sKlotho and smoking (Figure 4).

## Discussion

In this nationally representative sample of adults with DM aged > 40 years, a negative association was found between sKlotho levels and CKD prevalence in participants with hypertension but not in those without hypertension. Furthermore, an interactive effect between hypertension and sKlotho on CKD was found among participants with DM which indicated that hypertension and sKlotho deficiency were greater than the sum of individual effects on CKD prevalence. In addition, this study provides epidemiological evidence for related experimental studies.

Klotho is an essential component of FGF-23 receptor complex. When kidney function declines, Klotho deficiency is accompanied by increased circulating FGF-23 levels, suppressed 1,25-dihydroxyvitamin D levels, and hyperphosphataemia. Many evidence suggests that these alterations could promote the occurrence of hypertension [24,47–49].

Several previous studies have reported the relationship between sKlotho levels and CKD in patients with DM, which have been summarized on the **Table S4** in Supplements. However, general consensus on the clinical significance of sKlotho on CKD in patients with DM has not yet been reached. This discrepancy could be attributed to different target populations, relatively small sample size, and overlooked covariables, such as comorbidities. To clarify the relationship between sKlotho levels and CKD prevalence in individuals with diabetes, our study was based on a larger representative survey of the general population. Potential confounders of the correlation between sKlotho and CKD were carefully considered both statistically and clinically. Moreover, considering the effects of FGF23-klotho axis on hypertension, we paid a special attention on whether hypertension, which is also a common comorbidity of diabetes, is the factor that results in the controversial relation between sKlotho and DKD. Our results confirmed a negative association between sKlotho levels and CKD prevalence in adults with DM, which was observed only in participants with hypertension.

Factors that may regulate sKlotho levels remain not elucidated. Since the Klotho is primarily produced in the renal tubules, it is hypothesized that klotho production is reduced in renal failure. The circulating soluble klotho levels is regulated mainly through two aspects: (1) Majority soluble Klotho are derived from proteolytic cleaving of the membrane-bound protein (shedding). The sKlotho levels could be regulated through inhibiting or enhancing secretases mediating this proteolytic cleavage. For example, the action of insulin may increase these enzymes [50], while tissue inhibitors of metalloproteinases (TIMPs) could block these secretases [51]. (2) Klotho expression can be regulated by multiple transcription factors. For example, NF-κB, angiotensin II, and HMG-CoA reductase are inhibitors of Klotho transcription. Some drugs, such as anti-diabetic drugs, statin, vitD, and RAS-inhibitors can up-regulate the expression of Klotho [52,53]. So, the sKlotho level are regulated by a complex multi-pathway system. The effects of these regulating factors in different stage of CKD need further exploration.

In our study, among patients with both diabetes and hypertension, the prevalence of CKD is significantly higher than those with diabetes alone (40.88% vs 22.24%), and it is negatively associated with sKlotho levels. This may be due to sKlotho deficiency and hypertension have a synergistic effect on CKD prevalence. Existing studies indicate that decreased sKlotho can exacerbate kidney injury in diabetic patients through various mechanisms, such as pro-inflammation and oxidative stress, pro-fibrosis, vascular lesions, disturbance of metabolic modulation, abnormal cell apoptosis, and autophagy [54,55]. When hypertension occurs as another insult, related pathophysiological mechanisms such as hemodynamic changes, activation of RAAS system, injury of the endothelial system, and intraglomerular high pressure could further aggravate kidney trauma [56,57]. This synergism results in a significant association between sKlotho and CKD in patients with both diabetes and hypertension. On the contrary, in diabetic patients without hypertension, our results shows that there is no significant association between sKlotho levels and CKD prevalence, and the underlying mechanisms remains to be elucidated. Further longitudinal studies are needed to confirm the the modifying effects of hypertension on this association in diabetes patients.

Interestingly, as shown in Figure 2(B), we found that among participants without hypertension, sKlotho levels approximately > 1,400 pg/mL were associated with higher odds of CKD prevalence. This might be due to more anti-diabetic drugs were used. Previous study showed that the anti-diabetic drugs such as metformin, GLP-1-based-γ-aminobutyric acid (GABA), PPAR-γ, and insulin could up-regulate Klotho gene expression [58]. We compared the characteristics of participants without hypertension grouped by sKlotho level of 1,400 pg/mL in supplements Table S3. Indeed, patients with sklotho levels > 1,400 pg/mL have higher blood glucose levels, higher uACR and eGFR levels (Table S3). We speculate that their sKlotho levels might be up-regulated by using more anti-diabetic drugs or other unknown factors.

In our subgroup analyses, among participants with hypertension, when analyses were further stratified by smoking habits, the protective effect of high sKlotho levels on CKD was insignificant in current smokers. A prior study had reported that, compared with never-smokers, current and former smokers had a significantly increased risk of CKD [59]. Our results suggest that smoking may counteract the protective effects of sKlotho on CKD.

This study had some limitations. First, this cross-sectional study was inherently unable to infer causality from the observed associations and was only able to identify correlations, which is a significant limitation as it was unable to exclude the possibility of reverse causality and establish a temporal relationship between Klotho deficiency and the development of CKD. Future evaluation in realistic clinical practice or longitudinal studies is needed to offer stronger evidence for fully understanding the causal links between these variables. Second, similar to any epidemiological investigation, unmeasured confounding variables may affect the correlation between sKlotho and CKD. Third, despite including a large sample, the study population was limited to US residents, which limits the generalizability of the findings. Furthermore, the data were only available for one-time assessment of eGFR and albuminuria, which may contribute to recall and self-reporting bias. Therefore, the accuracy of our results may have been affected. Finally, the current study combined data from 5 cycles of NHANES survey, and quality control and measurement methods may differ among these cycles. However, because the NHANES was strictly conducted using a well-designed protocol, we believe that our findings are still reliable.

## Conclusions

Among individuals with DM, an signification association between sKlotho levels and CKD prevalence was observed only in those with hypertension. Hypertension modified the association between sKlotho levels and CKD prevalence. Both additive and multiplicative interactions were observed between hypertension and sKlotho on CKD. However, the causalities between hypertension, sklotho, and CKD in diabetic patients need further exploration, and underlying mechanisms warrants elucidation.

## Supplementary Material

Suplement.docx

## Data Availability

Data described in the manuscript, code book, and analytic code will be made available upon reasonable request at the NHANES website: https://www.cdc.gov/nchs/nhanes/index.htm.
