# Supplementary material for: Hypertension modifies the association between serum Klotho and chronic kidney disease in US adults with diabetes: a cross-sectional study of the NHANES 2007–2016
Source: Ren Fail. 2025 May 5;47(1):2498089. doi: 10.1080/0886022X.2025.2498089 (PMC12054556; doi:10.1080/0886022X.2025.2498089)
Supplement: Suplement.docx [file IRNF_A_2498089_SM3479.docx]

**Supplements**

Table S1. The distributions of covariables with missing data.

| **variable** | **Number of Missing data (N)** | **Percentage of missing data (%)** |
| --- | --- | --- |
| Gender | 0 | 0 |
| Age | 0 | 0 |
| Race | 0 | 0 |
| Marital status | 2 | 0.061 |
| Education level | 2 | 0.061 |
| Income level | 287 | 8.692 |
| Smoking habit | 3 | 0.091 |
| Drinking habit | 190 | 5.754 |
| Physical activity | 0 | 0 |
| BMI | 52 | 1.575 |
| CVD history | 0 | 0 |
| Serum albumin | 0 | 0 |
| Triglycerides | 2 | 0.060 |
| Cholesterol | 2 | 0.061 |
| Serum calcium | 1 | 0.030 |
| Serum phosphorus | 2 | 0.061 |
| Aspartate transaminase | 1 | 0.030 |
| Alanine transaminase | 1 | 0.030 |
| Pulse pressure | 265 | 8.025 |
| Glycated hemoglobin | 1 | 0.11 |
| Using RAS-inhibitors | 54 | 1.635 |
| Using statins | 52 | 1.575 |
| Using insulin | 44 | 1.3325 |

Abbreviations: BMI, body mass index; CVD, cardiovascular disease; RAS, renin-angiotensin-aldosterone system.

Table S2. Weighted multivariable logistic regression models evaluating the association between sKlotho and CKD (using multi-imputation data).

|  |  |  | **Non-adjusted Model** | | **Model 1** | | **Model 2** | | **Model 3** | | **Model 4** | |
| --- | --- | --- | --- | --- | --- | --- | --- | --- | --- | --- | --- | --- |
| Variable | n. total | n. event (%) | OR (95%CI) | *P*-value | OR (95%CI) | P-value | OR (95%CI) | *P*-value | OR (95%CI) | *P*-value | OR (95%CI) | *P*-value |
| **Group of hypertension** | |  |  |  |  |  |  |  |  |  |  |  |
| sKlotho < 806 pg/mL | 1,265 | 584 (46.5) | ref |  | ref |  | ref |  | ref |  | ref |  |
| sKlotho ≥ 806 pg/mL | 1,201 | 424 (35.3) | 0.63 (0.52, 0.76) | **<0.001** | 0.65 (0.52, 0.81) | **<0.001** | 0.64 (0.52, 0.80) | **<0.001** | 0.55 (0.43, 0.69) | **<0.001** | 0.55 (0.43, 0.70) | **<0.001** |
| **Group of non-hypertension** | | |  |  |  |  |  |  |  |  |  |  |
| sKlotho < 806 pg/mL | 385 | 84 (21.8) | ref |  | ref |  | ref |  | ref |  | ref |  |
| sKlotho ≥ 806 pg/mL | 451 | 102 (22.6) | 0.98 (0.70, 1.39) | 0.915 | 1.05 (0.75, 1.47) | 0.773 | 1.08 (0.77, 1.53) | 0.64 | 0.85 (0.57, 1.25) | 0.393 | 0.89 (0.60, 1.32) | 0.566 |

NOTES: Model 1 adjusts for age, gender, race, education level, marital status (have partner or no), and income level ; Model 2 adjusts for adjusts I +CVD history+drinking habits+smoking habits +physical activity +BMI; Model 3 adjusts for adjusts 2 + triglyceride+ total cholesterol+ serum calcium+ serum phosphorus + glycated hemoglobin+ ALT+ AST + pules pressure; Model 4 adjusts for adjusts 3 + using RAS-inhibitors + using statins + using insulin.

Table S3. Characteristics of Diabetic patients without hypertension grouped by sKlotho level (1400 pg/mL).

| **variables** | **Total (n=836)** | **sKlotho < 1400 pg/mL**  **(n=779)** | **sKlotho≥ 1400 pg/ml**  **(n=57)** |
| --- | --- | --- | --- |
| Glycated hemoglobin (%), Mean ± SD | 7.4 ± 1.9 | 7.3 ± 1.8 | 8.7 ± 2.8 |
| eGFR (mL/min/1.73 m^2^), Mean ± SD | 92.4±18.6 | 92.2±18.6 | 95.5±19.2 |
| Fasting blood glucose (mmol/L), Mean ± SD | 8.6± 3.6 | 8.5±3.4 | 10.8±5.3 |
| uACR (mg/g.Cr), Median[IQR] | 9.3 [5.3, 20.2] | 8.9 [5.3, 18.8] | 13.2 [6.8, 40.5] |
| Using RAS-inhibitors, n(%) | 208 (25.3) | 199 (25.9) | 9 (16.1) |
| Using insulin, n(%) | 110 (13.4) | 102 (13.3) | 8 (14.3) |
| CKD, n (%) | 186 (22.2) | 164 (21.1) | 22 (38.6) |
| Normoal-albuminuric CKD, n(%) | 34 (4.1) | 31 (4) | 3 (5) |
| Albuminuric CKD, n(%) | 152 (18.2) | 133 (17.1) | 19 (33.3) |

Table S4. Summary of existing studies on the association between Klotho and CKD

| **First author** | **Pub Year** | **subjects** | **Sample size** | **exposure** | **outcome** | **Study design** | **results** |
| --- | --- | --- | --- | --- | --- | --- | --- |
| [Kaixi Chang](https://pubmed.ncbi.nlm.nih.gov/?term=Chang+K&cauthor_id=36675565) [1] | 2023 | US adults aged 40-79 years | 13,584 | Serum klotho | Urinary albumin | cross-sectional study | A negative correlation between α-Klotho and urinary albumin was demonstrated. |
| [Zilong Zhang](https://pubmed.ncbi.nlm.nih.gov/?term=Zhang+Z&cauthor_id=36276390) [2] | 2022 | US adults aged 40-79 years | 13,589 | Serum klotho | CKD | cross-sectional study | Serum soluble Klotho levels were positively associated with eGFR and negatively associated with the prevalence of CKD |
| [David A Drew](https://pubmed.ncbi.nlm.nih.gov/?term=Drew+DA&cauthor_id=28104822) [3] | 2017 | US adults aged 70-79 years | 2,494 | Serum klotho | decline in kidney function, incidence of CKD | Cohort study | A higher serum klotho level was associated with a lower risk of decline in kidney function. 2) No significant association between serum Klotho and CKD incidence was found. |
| [Sudeep Jena](https://pubmed.ncbi.nlm.nih.gov/?term=Jena+S&cauthor_id=35812534) [4] | 2022 | 50 CKD patients, 40 healthy control | Total 90 | Serum klotho, oxidative stress | eGFR | case-control study | serum Klotho level was significantly higher in CKD patients than in healthy volunteers. 2) serum α-Klotho and oxidative stress were negatively correlated with eGFR in CKD patients |
| [Caihong Xin](https://pubmed.ncbi.nlm.nih.gov/?term=Xin+C&cauthor_id=35692408) [5] | 2022 | 2,055 patients with diabetes, 767 healthy controls, and 196 Diabetic nephropathy | total 3,018, (14 studies) | Serum klotho | Diabetic nephropathy | Meta-Analysis (observational study) | The sKlotho level in patients with diabetic nephropathy was significantly lower than that in diabetic patients without CKD, and was significantly lower in the early stage of diabetic nephropathy. |
| [Stefano Ciardullo](https://pubmed.ncbi.nlm.nih.gov/?term=Ciardullo+S&cauthor_id=35286490) [6] | 2022 | 2,989 patients with diabetes | 2,989 | eGFR | Serum klotho | cross-sectional study | eGFR were positively associated with s-Klotho. |
| Bob F [7] | 2019 | 63 patients with diabetic kidney disease | 63 | Serum Klotho | eGFR, annual decline of kidney function | Cohort study | The s-Klotho level did not correlate with eGFR, on average it was higher (but not significantly) in patients with eGFR below 60ml/min/1.73m.  There was a strong correlation of s-Klotho with the rate of reduction of eGFR/year. |
| [A Inci](https://pubmed.ncbi.nlm.nih.gov/?term=Inci+A&cauthor_id=27466997) [8] | 2016 | 109 patients with early stage of diabetic nephropathy , 32 healthy controls | Total 141 | Serum Klotho | eGFR | cross-sectional study | There was no correlation between eGFR and s-Klotho levels. |
| [Panagiotis Savvoulidis](https://pubmed.ncbi.nlm.nih.gov/?term=Savvoulidis+P&cauthor_id=32274109) [9] | 2020 | 30 patients with CKD stage V, 30 patients with CKD stage III | Total 60 | Serum Klotho | Calcification of aortic valve and coronary arteries | cross-sectional study | a-Klotho decrease with worsening CKD severity but no correlation was found between the levels of a-Klotho and severity of coronary arteries and aortic valve calcification |
| [Zuzanna Gamrot](https://pubmed.ncbi.nlm.nih.gov/?term=Gamrot+Z&cauthor_id=34647605) [10] | 2021 | 42 children with CKD, 21 healthy control children | 63 | Serum Klotho, | serum creatinine, eGFR. | cross-sectional study | A significant positive correlation was found between serum serum αKLotho concentration and serum creatinine, but no correlation between serum αKLotho and eGFR. |
| [Atma Gunawan](https://pubmed.ncbi.nlm.nih.gov/?term=Gunawan+A&cauthor_id=34035901) [11] | 2020 | - | - | klotho gene polymorphisms | CKD | systematic review (observational study) | KL G395A were found to correlate with increased susceptibility to CKD. |
| [Yan Guo](https://pubmed.ncbi.nlm.nih.gov/?term=Guo+Y&cauthor_id=38818966) [12] | 2024 | 65 CKD patients | 65 | Renal α-klotho levels | eGFR | cross-sectional study | Renal α-klotho levels are associated positively with eGFR. |
| [QiFeng Liu](https://pubmed.ncbi.nlm.nih.gov/?term=Liu+Q&cauthor_id=34483963) [13] | 2021 | CKD patients | 1,204 | Serum Klotho | vascular calcification | Meta-Analysis (observational study) | a significant association between decreased sKlotho level and increased risk of VC in CKD patients was found. |
| [Zhongyu Fan](https://pubmed.ncbi.nlm.nih.gov/?term=Fan+Z&cauthor_id=38396063) [14] | 2024 | CKD patients | 1,944 | Serum Klotho | serum calcium , phosphorus | Meta-Analysis (observational study) | a significant positive correlation between sKlotho and Ca and a remarkable negative connection between sKlotho and P were found. |

References:

1. [Kaixi Chang](https://pubmed.ncbi.nlm.nih.gov/?term=Chang+K&cauthor_id=36675565) , [Yupei Li](https://pubmed.ncbi.nlm.nih.gov/?term=Li+Y&cauthor_id=36675565), [Zheng Qin](https://pubmed.ncbi.nlm.nih.gov/?term=Qin+Z&cauthor_id=36675565), [Zhuyun Zhang](https://pubmed.ncbi.nlm.nih.gov/?term=Zhang+Z&cauthor_id=36675565), [Liya Wang](https://pubmed.ncbi.nlm.nih.gov/?term=Wang+L&cauthor_id=36675565) ,et al. Association between Serum Soluble α-Klotho and Urinary Albumin Excretion in Middle-Aged and Older US Adults: NHANES 2007-2016. J Clin Med. 2023 Jan 13;12(2):637.

# 2) [Zilong Zhang](https://pubmed.ncbi.nlm.nih.gov/?term=Zhang+Z&cauthor_id=36276390), [Xianghong Zhou](https://pubmed.ncbi.nlm.nih.gov/?term=Zhou+X&cauthor_id=36276390), [Linghui Deng](https://pubmed.ncbi.nlm.nih.gov/?term=Deng+L&cauthor_id=36276390), [Kun Jin](https://pubmed.ncbi.nlm.nih.gov/?term=Jin+K&cauthor_id=36276390), [Xingyu Xiong](https://pubmed.ncbi.nlm.nih.gov/?term=Xiong+X&cauthor_id=36276390), et al. The association between serum soluble Klotho and chronic kidney disease among us adults ages 40 to 79 years: Cross-sectional study. Front Public Health. 2022 Oct 6:10:995314.

3) [David A Drew](https://pubmed.ncbi.nlm.nih.gov/?term=Drew+DA&cauthor_id=28104822), [Ronit Katz](https://pubmed.ncbi.nlm.nih.gov/?term=Katz+R&cauthor_id=28104822), [Stephen Kritchevsky](https://pubmed.ncbi.nlm.nih.gov/?term=Kritchevsky+S&cauthor_id=28104822), [Joachim Ix](https://pubmed.ncbi.nlm.nih.gov/?term=Ix+J&cauthor_id=28104822), [Michael Shlipak](https://pubmed.ncbi.nlm.nih.gov/?term=Shlipak+M&cauthor_id=28104822), et al. Association between Soluble Klotho and Change in Kidney Function: The Health Aging and Body Composition Study. J Am Soc Nephrol. 2017 Jun;28(6):1859-1866.doi: 10.1681/ASN.2016080828.

1. [Sudeep Jena](https://pubmed.ncbi.nlm.nih.gov/?term=Jena+S&cauthor_id=35812534), [Pratikhya Sarangi](https://pubmed.ncbi.nlm.nih.gov/?term=Sarangi+P&cauthor_id=35812534), [Upendra K Das](https://pubmed.ncbi.nlm.nih.gov/?term=Das+UK&cauthor_id=35812534), [Andrew A Lamare](https://pubmed.ncbi.nlm.nih.gov/?term=Lamare+AA&cauthor_id=35812534), [Roma Rattan](https://pubmed.ncbi.nlm.nih.gov/?term=Rattan+R&cauthor_id=35812534),et al. Serum α-Klotho Protein Can Be an Independent Predictive Marker of Oxidative Stress (OS) and Declining Glomerular Function Rate in Chronic Kidney Disease (CKD) Patients. Cureus. 2022 Jun 8;14(6):e25759.
2. [Caihong Xin](https://pubmed.ncbi.nlm.nih.gov/?term=Xin+C&cauthor_id=35692408), [Xin Sun](https://pubmed.ncbi.nlm.nih.gov/?term=Sun+X&cauthor_id=35692408), [Zheng Li](https://pubmed.ncbi.nlm.nih.gov/?term=Li+Z&cauthor_id=35692408) , [Tianshu Gao](https://pubmed.ncbi.nlm.nih.gov/?term=Gao+T&cauthor_id=35692408). Relationship of Soluble Klotho and Early Stage of Diabetic Nephropathy: A Systematic Review and Meta-Analysis. Front Endocrinol (Lausanne) . 2022 May 27:13:902765.
3. [Stefano Ciardullo](https://pubmed.ncbi.nlm.nih.gov/?term=Ciardullo+S&cauthor_id=35286490), [Gianluca Perseghin](https://pubmed.ncbi.nlm.nih.gov/?term=Perseghin+G&cauthor_id=35286490). Soluble α-Klotho levels, glycemic control and renal function in US adults with type 2 diabetes. Acta Diabetol. 2022 Jun;59(6):803-809.
4. Bob F, Schiller A, Timar R, Lighezan D, Schiller O, et al. Rapid decline of kidney function in diabetic kidney disease is associated with high soluble Klotho levels. Nefrologia. 2019;39 (3): 250–257.
5. [A Inci](https://pubmed.ncbi.nlm.nih.gov/?term=Inci+A&cauthor_id=27466997), [F Sari](https://pubmed.ncbi.nlm.nih.gov/?term=Sari+F&cauthor_id=27466997), [R Olmaz](https://pubmed.ncbi.nlm.nih.gov/?term=Olmaz+R&cauthor_id=27466997), [M Coban](https://pubmed.ncbi.nlm.nih.gov/?term=Coban+M&cauthor_id=27466997), [S Dolu](https://pubmed.ncbi.nlm.nih.gov/?term=Dolu+S&cauthor_id=27466997), et al. Soluble Klotho levels in diabetic nephropathy: relationship with arterial stiffness. Eur Rev Med Pharmacol Sci. 2016 Jul;20(15):3230-7.
6. [Panagiotis Savvoulidis](https://pubmed.ncbi.nlm.nih.gov/?term=Savvoulidis+P&cauthor_id=32274109), [Andreas P Kalogeropoulos](https://pubmed.ncbi.nlm.nih.gov/?term=Kalogeropoulos+AP&cauthor_id=32274109), [Vasileios Raptis](https://pubmed.ncbi.nlm.nih.gov/?term=Raptis+V&cauthor_id=32274109), [Vasileios Rafailidis](https://pubmed.ncbi.nlm.nih.gov/?term=Rafailidis+V&cauthor_id=32274109), [Panagiotis I Georgianos](https://pubmed.ncbi.nlm.nih.gov/?term=Georgianos+PI&cauthor_id=32274109), et al. Calcification of coronary arteries and aortic valve and circulating a-klotho levels in patients with chronic kidney disease. J Thorac Dis. 2020 Mar;12(3):431-437.
7. [Zuzanna Gamrot](https://pubmed.ncbi.nlm.nih.gov/?term=Gamrot+Z&cauthor_id=34647605), [Piotr Adamczyk](https://pubmed.ncbi.nlm.nih.gov/?term=Adamczyk+P&cauthor_id=34647605), [Elżbieta Świętochowska](https://pubmed.ncbi.nlm.nih.gov/?term=%C5%9Awi%C4%99tochowska+E&cauthor_id=34647605), [Dagmara Roszkowska-Bjanid](https://pubmed.ncbi.nlm.nih.gov/?term=Roszkowska-Bjanid+D&cauthor_id=34647605). Tumour necrosis factor alpha (TNFα) and alpha-Klotho (αKL) in children and adolescents with chronic kidney disease (CKD). Endokrynol Pol. 2021;72(6):625-633.
8. [Atma Gunawan](https://pubmed.ncbi.nlm.nih.gov/?term=Gunawan+A&cauthor_id=34035901), [Jonny Karunia Fajar](https://pubmed.ncbi.nlm.nih.gov/?term=Fajar+JK&cauthor_id=34035901), [Fredo Tamara](https://pubmed.ncbi.nlm.nih.gov/?term=Tamara+F&cauthor_id=34035901), [Aditya Indra Mahendra](https://pubmed.ncbi.nlm.nih.gov/?term=Mahendra+AI&cauthor_id=34035901). Nitride oxide synthase 3 and klotho gene polymorphisms in the pathogenesis of chronic kidney disease and age-related cognitive impairment: a systematic review and meta-analysis. F1000Res. 2020 Apr 9:9:252.

1. [Yan Guo](https://pubmed.ncbi.nlm.nih.gov/?term=Guo+Y&cauthor_id=38818966), [Feng Wan](https://pubmed.ncbi.nlm.nih.gov/?term=Wan+F&cauthor_id=38818966), [Yan-Peng Shi](https://pubmed.ncbi.nlm.nih.gov/?term=Shi+YP&cauthor_id=38818966) , [Hong-Mei Zhang](https://pubmed.ncbi.nlm.nih.gov/?term=Zhang+HM&cauthor_id=38818966), [Ru-Chun Yang](https://pubmed.ncbi.nlm.nih.gov/?term=Yang+RC&cauthor_id=38818966). Association between renal α-klotho and renal pathology among patients with chronic kidney disease. Ther Apher Dial. 2024 Oct;28(5):769-774.
2. [QiFeng Liu](https://pubmed.ncbi.nlm.nih.gov/?term=Liu+Q&cauthor_id=34483963), [LiXia Yu](https://pubmed.ncbi.nlm.nih.gov/?term=Yu+L&cauthor_id=34483963), [XiaoYa Yin](https://pubmed.ncbi.nlm.nih.gov/?term=Yin+X&cauthor_id=34483963), [JianMing Ye](https://pubmed.ncbi.nlm.nih.gov/?term=Ye+J&cauthor_id=34483963), [ShaSha Li](https://pubmed.ncbi.nlm.nih.gov/?term=Li+S&cauthor_id=34483963). Correlation Between Soluble Klotho and Vascular Calcification in Chronic Kidney Disease: A Meta-Analysis and Systematic Review. Front Physiol 2021 Aug 13:12:711904.

1. [Zhongyu Fan](https://pubmed.ncbi.nlm.nih.gov/?term=Fan+Z&cauthor_id=38396063), [Xuejiao Wei](https://pubmed.ncbi.nlm.nih.gov/?term=Wei+X&cauthor_id=38396063), [Xiaoyu Zhu](https://pubmed.ncbi.nlm.nih.gov/?term=Zhu+X&cauthor_id=38396063), [Kun Yang](https://pubmed.ncbi.nlm.nih.gov/?term=Yang+K&cauthor_id=38396063), [Ling Tian](https://pubmed.ncbi.nlm.nih.gov/?term=Tian+L&cauthor_id=38396063), et al. Correlation between soluble klotho and chronic kidney disease-mineral and bone disorder in chronic kidney disease: a meta-analysis.Meta-Analysis Sci Rep. 2024 Feb 23;14(1):4477.
